# Commissioning simulations to test new healthcare facilities: a proactive and innovative approach to healthcare system safety

**DOI:** 10.1186/s41077-019-0107-8

**Published:** 2019-07-16

**Authors:** Alyshah Kaba, Sue Barnes

**Affiliations:** 1 eSIM Provincial Program, Alberta Health Services, South Tower Foothills Hospital, 1403 29 St. NW, Calgary, T2N 2T9 Canada; 2 Department of Community Health Sciences, Cumming School of Medicine, University of Calgary, Foothills Medical Centre, 1403 29th Street NW, Calgary, AB T2N 2 T9 Canada; 3 South eSIM Provincial Simulation Program, Alberta Health Services, South Health Campus, 4448 Front Street SE, Calgary, T3M 1 M4 Canada

**Keywords:** In situ, Commissioning, Simulation, New healthcare facilities, Process-orientated simulations, System integration, Latent safety threats, Patient safety, Healthcare systems, innovation

## Abstract

Development and reconstruction of new healthcare facilities and spaces has the potential for latent safety threats to emerge, specifically unintentional harm that could affect actual patients once the facility opens, such as missing equipment, inefficient setup, or insufficient space for procedures. Process-orientated simulation and testing is a novel innovation in healthcare. The aim of process-orientated simulations and debriefing is to examine the process of care, rather than the outcome of care. These simulations, which take place in actual patient care settings and environments prior to occupancy, are an emerging strategy that can be used to test new environments and new healthcare facilities to ensure that the spaces created match the needs of the staff and administration, while proactively identifying latent safety threats prior to delivering patient care. In turn, these simulations can be also be used as part of the new site orientation and training plan. The aim of this paper is to examine a case study describing the use of the novel innovation of process-orientated simulations to test the opening of a new 300-bed healthcare facility.

## Introduction

In North America, approximately 1340 healthcare medical construction projects are currently in progress [1]. A 2016 Hospital Construction Survey identified $97 billion was being invested into new hospitals, expansions, off-campus clinic, and medical offices [1]. Along with aging infrastructure, many more hospital units are being redesigned and restructured to accommodate increased patient loads, innovative technologies, and new ways of caring for patients in healthcare [2, 3].

During the planning and construction phase of any new healthcare facility, there must be an integration of the perspectives of the architects, engineers, project management teams, and quality team members along with the operational requirements from leadership and the clinical requirements from clinicians who are at the front line of patient care [4]. Relevant theoretical underpinning of research from human factors applied to healthcare, such as the Systems Engineering Initiative for Patient Safety (SEIPS) model, has provided a framework for recognizing core principles in system design [5]. Further expansion of the SEIPS 2.0 model focuses on the work system, consisting of the organization, internal/external environment, tasks, tools, technology and person(s), the processes, and outcomes [5, 6]. These factors are ultimately critical in the construction or redesign of any new healthcare building.

Simulation-based education has historically focused on individual training for healthcare learners and team training for practicing professionals [7–9]. Process-orientated simulation is an emerging field in healthcare which uses simulation to examine the process of care, rather than the outcome of care [10]. While process-oriented simulations can include system integration simulation, process simulations focus on gaining and providing information collected from simulation sessions for individual leadership to act upon when safety issues are identified. The concept of system integration is a much broader engineering term which relates to bringing together the component of subsystems into one system that functions together. Moreover, in healthcare, system integration is the ability to improve the quality of care and patient outcomes through re-engineering of care delivery processes [12].

For example, using simulation to re-create an emergency-based patient encounter within their intrinsic environment could reveal latent safety threats such as poor availability of patient equipment, inadequate emergency call buttons, or unsafe obstacles [11–14]. Latent safety threats are defined as unintentional harm that could affect actual patients once the facility opens, such as missing equipment, inefficient setup, or insufficient space for procedures. These process-orientated simulations which take place in an actual patient care setting and environments prior to occupancy can be used as a method for ensuring that the spaces created match the needs of the staff and administration while proactively identifying latent safety threats prior to first patient care [14, 15]. By only theorizing about the care delivery process, unintentional harm could affect actual patients once the facility opens. The application of simulation training within the actual patient care environment, referred to as in situ sessions, has become an exciting option, achieving the highest level of realism [15–22].

In the last decade, there has been emerging literature that looks at the use of process-oriented simulation training for the opening of new and renovated units, construction, and opening of new hospitals. Most examples have focused on openings of specific units within existing spaces and some within new satellites/wings in the hospital. Examples described in the literature include established teams and transference of established policies, protocols, and equipment to the new space from the legacy site [23–37].

In other regulated industries such as the construction industry, building commissioning is part of the quality assurance process provided for Facilities Maintenance and Engineering (FM&E) staff during and following construction. The process assures non-clinical FM&E staff and leadership that all systems and components of a building or industrial plant are designed, installed, tested, operated, and maintained according to the operational requirements of the owner or final client [25].

Unfortunately, in comparison with healthcare, such requirements are non-existent for handing over of new infrastructure for clinical staff who will be providing care to patients in these new areas, often leaving training and new systems/processes untested until the first patient arrives, which is a potential risk for patient safety.

Simulation for commissioning new environments, through the development of process-orientated simulations, is an innovative approach to test these processes and new systems to ensure safe facilities, safe patient care, and recognition of latent safety threats that may prevent delays in opening and decrease costs in reconstruction [29]. In turn, these simulations could also be expanded for use as a part of the new site’s orientation and training plan. The following paper will explore a case study describing the use of process-orientated simulations to test the opening of a new 300-bed healthcare facility.

## Case study of simulation innovation

South Health Campus (SHC) is one of the largest hospitals in Alberta, Canada, with a footprint roughly the same size as a large commercial mall. Construction began in 2007 for phase 1 for a 300-bed facility consisting of inpatient and outpatient services. The phased opening of this newly constructed acute care hospital (258 inpatient beds and 66 outpatient clinics) started in July of 2012. Onboarding hundreds of new staff for each area/department involved a general site familiarization, including wayfinding, new department orientations, vendor training, and construction safety training, as the site in the early stages was still being considered under control of the construction firm. Final occupancy for the last clinical unit was accomplished in September 2014, at an approximate cost of 1.3 billion Canadian dollars (CAD).

As part of this unique prospect for the opening of this new hospital, Alberta Health Services (AHS) Provincial Simulation Program, eSIM, developed a comprehensive in situ simulation project that included both clinical and non-clinical areas. In situ simulations took place in patient care settings, public spaces, support services, and administration areas in an effort to achieve the most realistic experience in evaluating the clinical environment prior to patient arrival to evaluate functionality, assess system processes, and identify areas of potential patient safety concern.

### Human resources

In order to facilitate and coordinate simulations for the site, two positions were created: a simulation consultant with clinical background and advanced simulationist training and a simulation technician with a clinical engineering background. These dedicated resources were essential for placing simulation in the forefront for commissioning of this new space. The support of a simulation technician allowed for set up and maintenance of all equipment for simulation. The simulation consultant acted as the liaison for engaging management and leadership in using simulation as a quality assurance and training strategy. Meetings with clinicians and leadership took place 1 year prior to the phased openings. Creation of department and clinical area specific cases was completed with medical vetting for specific cases. All simulations were run by a team of simulation specialists trained in advanced debriefing techniques, alongside clinicians and physicians with expertise in their practice areas. Over 50 clinicians representing varying clinical and non-clinical areas, allied health, and physicians were trained as facilitators for their simulations through the AHS eSIM Workshop in Simulation Education (WISE ™1) 1 course. This interactive and immersive 2-day course allowed individuals to become independent users of simulation, from scenario design, briefing, facilitating, and debriefing skills. As part of the extensive mentorship, faculty were paired with the SHC simulation consultant and acted both as a new simulationist and as the content expert for the areas that they were supporting in the new opening of their departments.

### Financial burden

The yearly cost of the 2 sponsored positions of simulation consultant and simulation technician was approximately $95,000 Canadian dollars (CAD) and $88,000 CAD gross, and although initially assigned to the site for commissioning, they continue to be responsible for zonal and provincial commitments for the larger program today. Expenditures for simulation lab mannequins used within the simulations (interactive patient simulators adult male, parturient, newborn, premie, and 2 pediatric (1 and 5 year olds) were covered through Calgary Health Trust foundational grants for approximately $225, 000 CAD in 2012. Training of SHC faculty in simulation education was at no fee for a provincial 2-day WISE ™1 course that provides a broad overview of core simulation concepts and principles to novice and intermediate educators and physicians. Department equipment was used for in situ simulation, and supplies that were opened were able to be re-used and repurposed for education, as all simulations took place without risk of exposure to real patients being present. The simulation training occurred as part of the orientation budget assigned to each unit, although physician participation was voluntary. Other relationships, such as the AHS Human Factors division, were consulted on an as need basis for various other projects at SHC, but did not create any additional cost for the commissioning simulations. Volunteer services provided standardized family members at no cost.

### Participants

**Fig. 1:**
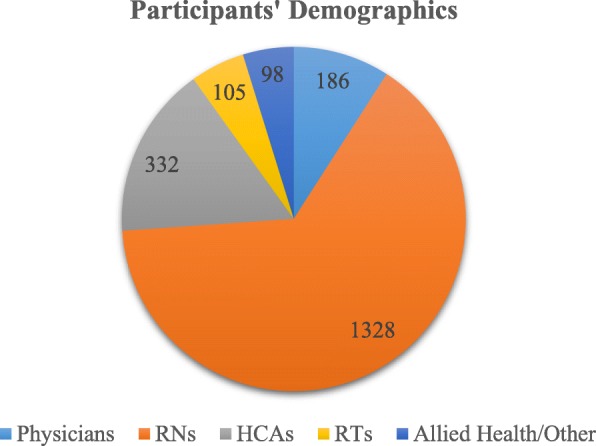
Participants’ Demographics (*n* = 2049). Note: exact numbers include multiple participation documented for several scenarios by the same staff

From a human resource perspective, new teams were being formed for all areas of this hospital. Participants in the simulation were newly hired health care providers, with varied years of experience. Challenges included large numbers of hires of out of zone, province, and country, as well as a higher proportion of new graduates, entering the workforce for the first time. In total, 186 physicians, 1328 registered nurses (RNs), 332 healthcare aides (HCAs), 105 respiratory therapists (RTs), and 98 allied health professionals participated in the simulation team training pre-opening (see Fig. 1).

### Procedure

In situ simulation sessions were co-developed with clinicians from each area, focusing on specific patient population scenarios evaluating “Day in the Life” type activities and on responding to crises that might occur within their area. Simulations incorporated new knowledge gained during general orientation such as specifics of the building (wayfinding), equipment, and vendor training. Sessions reflected similar numbers of participants representing real healthcare teams, as an example, having 3–4 healthcare professionals such as the RN, RT, and nurse practitioner for an ICU admission, versus larger numbers to represent interdepartmental teams responding to a site wide event (Pediatric Code Blue in clinic setting with ED and ICU team response). Number of sessions run per area varied if the focus was on a system-wide exercise (Code Green Evacuation, Power Failure, and Code Red (Fire) or multiple sessions for unit orientations. See area-specific simulations—Table 1).

**Table 1:** Area-specific simulations

Opening date	Area	Theme of scenarios	Number of scenarios completed/participants
August 2012	Clinical support services (FM&E, housekeeping, supply, administration, lab, clinical engineering, protection services)	First simulations were done incorporating those outside clinical services using an advanced first responder/Code Blue event. No clinical support 911 response required. Most departments had limited first aide responders only	7 days/14 sessions 124 participants
September 2012 May 2013	Emergency disaster management	Code Green (evacuation), Code Red (fire)/loss of power, Code White (aggressive patient), Code Yellow (missing Marvin Site response)	1 session—65 participants 1 session—59 participants 1session—44 participants 1 session—68 participants
	Diagnostic imaging Magnetic resonance imaging	Anaphylaxis due to contrast injection Code Blue events in MRI and CT area requiring a 911 response to site Way finding becomes a major issue - collaborations for EMS crews with protection services as a key player	3 days—6 Sessions 42 participants 1session—34 participants
	Neuro outpatient clinics/rehab	Seizure	5 days—10 sessions 68 participants
January 2013	Emergency department	Trauma, acute coronary syndrome requiring transfer to catheterization lab at another hospital, precipitous birth, pediatric asthma attack	3 days multiple concurrent sessions—48 sessions 240 participants
February 2013	Intensive care unit (ICU)	Code Blue response all units, Code 66 – medical emergency team, anaphylaxis in a public area, transfer to operating room	Multiple days concurrent with orientation—24 sessions 96 participants
April 2013	Medical inpatient units and outpatient clinics	Code Blue	Multiple days concurrent with orientation—138 sessions 690 participants
	Operating rooms (OR) Post-anesthetic care	Code Blue, malignant hyperthermia, trauma, transfer from ED to OR to ICU	4 days—12 sessions 144 participants 2 days—6 sessions 19 participants
July 2103	Pediatric outpatient clinics	Seizure, asthma	2 sessions—28 participants
September 2103	Family maternal practice (FMP) Neonatal intensive care unit (NICU) Women’s Clinic	Vaginal birth with vacuum/forceps, cord prolapse with transfer to OR, post-partum hemorrhage, neonatal resuscitation, maternal code	12 days—multiple concurrent sessions— 108 sessions 282 participants
July 2014	Cardiac intensive care unit (CICU)	Acute coronary syndrome, Code Blue, extreme bradycardia requiring pacing, synchronized cardioversion	1 day—4 sessions/16 participants

## Findings

### Debriefings

All simulations were performed in the actual care environments using the personnel, equipment, medications, and resources intended for clinical care. A formalized debriefing was conducted after every simulation following the Promoting Excellence And Reflective Learning in Simulation (PEARLS) framework utilizing common debriefing strategies such as plus/delta, focused facilitation, and directive feedback [36–42]. At the time, focus for commissioning simulations centered on the system issues versus individual performance within a medical scenario. Much like the elements identified in the SEIPS, framework for healthcare design themes emerged with each simulation [5, 6]. The work system simulations identified gaps and deficiencies, such as tools (missing equipment and supplies), human resources/persons (understanding of roles, composition of teams), communication/technology (nurse call system, Vocera, paging, cell/telephone services), organizational (deficiencies from build), processes (room layouts), and transport routes. After each simulation, debriefing and documentation of findings were collected and reviewed by leadership teams in each area to assign operational ownership and responsibility. Environmental latent safety threats identified were completed prior to opening. Human resource recommendations on team composition, roles and responsibilities, and scope of practice were brought back and further explored over the first year of opening, with subsequent simulations being run for evaluation of changes.

### Examples of large-scale simulation findings

With the phased opening approach of various clinical units and departments, the arrival of different support and clinical teams differentiated the responses that were available to the personnel occupying the building. For example, the site did not have an active emergency department until 4 months after the opening of the ambulatory clinics. This required simulations to incorporate a community call out for 911 services as part of their emergency response plan for a patient deterioration in the building. Internally, the organization and occupying personnel had the strength as healthcare professionals to handle some first aide responses but needed to rely on external support of city emergency services. One large-scale simulation event for the diagnostic imaging department revealed that emergency medical services’ (EMS) response to the call failed to find the front entrance of the hospital. Assumptions had been made by EMS to show up at the ambulance bays in the ED, which were not functional at the time. Debriefings identified this knowledge barrier, and tours/orientations for all EMS providers were arranged in the following months. Role clarity was also recognized for the protection services personnel as an important way-finder for EMS once they arrived on site. As per the SEIPS model, recognition of system deficiencies identified limitations in the internal environment, barriers with external support such as EMS, the process that needed to be changed in order to access help and ultimately the outcome for staff and patient safety [5, 6].

Further internal obstacles came once the emergency department opened, as there was no obstetrical services on site for 9 months. This prompted simulation training to include a potential precipitous birth. Debriefings identified a need for targeted neonatal resuscitation certification for all nursing staff and simulations surrounding other obstetrical crises such as a post-partum hemorrhage. A workflow process for transferring a laboring mom, or a mother and neonate who delivered on site, to a hospital site with maternity-post-partum services required coordination using zonal healthcare services RAAPID (Referral, Access, Advice, Placement, Information & Destination) and EMS. Creation of a patient transfer process and teaching of zonal resources were then disseminated to emergency staff.

One large-scale exercise looked at external and internal coordination of hospital and Emergency Services (Fire Department). This event involved a simulation Code Red (Fire) and simultaneous power outage. The site has their family maternity place (labor and delivery/post-partum/neonatal intensive care areas) located on the seventh floor, and the surgical suites were located on the third floor. Any potential caesarean sections need to be carried out on the third level, utilizing a dedicated elevator with badge swipe access for retrieval and operation. During a Code Red event, the facility’s technology homed the elevators to the main level. The work system as designed failed during the simulation. Debriefings identified the need to be able to evacuate and transfer a laboring patient in crisis from the labor unit to the operating theatres. Discussions also poised that a similar case of cord prolapse, with a healthcare provider performing a medical intervention to maintain perfusion to the neonate, may not allow a transfer to be possible. Process changes to the organization’s care plans looked towards solutions. Purchase of evacuation sleds for patients for use on stairs was obtained. Internal tasks included a new surgical case cart for a cesarean section to be kept on the seventh floor in case a transfer was not medically possible.

A few examples of detailed discoveries and recommendations are highlighted in Table 2. Simulation session numbers were based upon the size of the department (some small clinics with matching small numbers of staff, some large departments with large staffing numbers, as well as interdependence between units (simulations covered department-specific needs and handovers of care between units such as ED to OR). With a phased approach to opening, some sessions were overlapping with other areas, with a small impact on the simulation resources at hand, which may have affected the consistency of numbers of sessions delivered. Areas that were not listed in the table to have found specific “recommendations” might have been due to the fact that findings from simulations were small and lessons learned may have been from a staff orientation and training benefit versus a gap or latent safety threat. These findings are not exhaustive of all deficiencies revealed. While these findings are specific to SHC, different institutions may learn from these themes to build their own simulations.

**Table 2:** Summary of findings and recommendations

Element/area	Findings	Recommendations	Actioned
Provider/team issues provider Roles and scope of practice Emergency department (ED) Med Surg OB units	• Trauma room/code team formation physicians, respiratory therapist and paramedics in code room, overlapping skill set and roles. • Team composition—addition of health care aid (unregulated) to units lack of understanding of scope (too much to little), partnership with RNs, lack of acute care hospital experience.	• Simulation teamwork training for identifying leader, role clarity, and communication. • Simulation teamwork training/orientation classroom for role clarity and communication.	• First year post-opening focused on team simulations. Elimination of paramedic role • Monthly simulation with IP teams continue in trauma bays • OB Sims monthly nursing
	• No OB on site until September 2014/ ED open January 2013.	• Expand NRP training to ED staff. • Need for precipitous delivery equipment and supplied in ED staff.	• OB orientation day for ED occurred December 2012 • Equipment and supplies arrived prior to opening
Clinical proficiencies Inpatient units ICU code team	• Medications—code team unable to access automatic dispensing cabinets on units. • No crash carts/defibrillators/code team prior to January 2013.	• Orientate nursing staff on role in accessing meds for code team. • Development of airway buckets pre ICU with AED training for staff. • Protocols placed in high acuity areas	• Completed • Airway buckets in effect from 2012 (dismantled with full operation of ICU operational in 2013) • Signage/resources created
Facility issues All units OB	• Code Red/Blue/power outage outside of fire department override during Code Blue; code team over team to use the stairs. • Dedicated OB elevators/OB 7th floor OR on 3rd floor	• Awareness, key to be given to facilities management • ID need to transfer sled to transport OB patients in need of STAT C-section/OR resuscitation • Need for C-section set up on 7th floor	• Key obtained • Site wide fire drills maintained yearly • Transfer sleds obtained • Confidence in elevators/system—not completed
Communication Inpatient units ICU code team Public areas	• Mis-wiring of Code Blue/staff assist buttons • No cell service/outside telephones in hallways for calls on site prior to mid-August 2012	• Immediate follow-up with vendor and facilities management • Staff awareness campaign for accessing hard-wired phones locations	• Wirings fixed and tested prior to opening • New phone lines for main street kiosks
Unintended consequences EMS	• EMS not aware of how to access hospital for pre ED opening	• Tours for all Calgary EMS providers to site	• Completed by October 2012
Emergency department	• Code room setup, pillars hinder access to med cupboards.	• Reconfiguration of carts and trauma room to better serve needs	• Re-configuring and changing of supply carts and resources completed
Adolescent mental health	• Asphyxiation/hanging of mannequin in simulation accomplished in high observation unit	• Management and staff awareness for need to constant observation, patient placement	• Grates fixed by FM and E, staffing and patient assignments changed
Pediatric outpatient clinic	• Pediatric Code Blue lack of pediatric supplies for code blue. No medications available in clinics, lack of specialty knowledge for pediatric crisis	• Identified need to “pack and go” to trauma bay in ED ASAP, meds added to RT outreach bag, stretcher brought to unit for potential transfers	• New pediatric backpack and supplies • Assigned situational role to ICU nurse to recommend when transfer needs to occur

## Discussion

### Partnerships

SHC opened with four foundational pillars: innovation, collaborative practice, wellness, and patient family-centered care, creating a culture where innovative strategies, such as simulation training, proved instrumental in establishing trust and openness for our teams to learn together. Collaborative practice allowed new relationships to develop, not only between clinical areas but also with non-clinical areas, such as protection services, emergency disaster management, and facilities maintenance engineering. Successes included protection services personnel building on SHC’s collaborative care pillar with their team’s responses to the mental health units. They introduced a “Patient First Strategy” to decrease “hands on” patients during escalating events. Debriefings following the simulations highlighted the special skill set and training the protective services responders have for dealing with patients in crisis. Consequently, new values were gained by clinical staff through their realization that these key support services are an integral part of the larger healthcare team.

Another unique unintended outcome was the lessons learned from the engagement of families (and potential patients) through simulation. Patient family-centered care members along with volunteer resources had members of the community act in the role as family and participated in debriefings, allowing them to experience how health professionals train. Feedback from participants highlighted how important it was to have the family member present during crisis events and identify the role of the patient family care support person [43, 44]. A practice support document guideline was developed emphasizing best practices of families as full partners in care. Volunteers are still an important part of the simulations to date, acting as standardized patients for domestic violence scenarios, abusive/aggressive patients, and patients for mass casualty incidents. One volunteer stated:

I feel honored to have helped these healthcare professionals out in the opening of this new hospital. It’s amazing to see that they are training together for emergencies. They listened to me as a family member when I had concerns in the debriefings.

Citizen Advisory Team Council Member and Volunteer for Simulation

### Sustaining results

Using inter-professional simulation to open the facility has resulted in a culture where simulation training has become a normal expectation for staff, educators, and management. These continuing sessions have reflected a more traditional use of medical simulation for intra-professional team training, staff orientations, certifications, and yearly competency assessments. Post site opening simulations from 2015 to 2018, 1336 sessions have been completed and 9352 participants have taken part in simulation activities.

The momentum within the institution continues to grow with larger system simulation and debriefing sessions conducted from 2014 to 2018 which focused on the incorporation/expansion to zonal training and testing of Emergency Disaster Management Codes including Code Orange (mass casualty), Code Brown (hazardous material – chemical decontamination), Code Yellow (missing person), Code White (aggressive patient/family), Code Purple (hostage), Code Red (fire), and EBOLA preparation (2014 and 2018).

Educational support for both the original educator/physician faculty and educators continues today through zonal simulation courses and conferences.

## Lessons learned

Simulation involvement in the early design and construction phase would have been a critical phase for involvement. Opportunities to work through tabletop simulations and mockups could have identified spaces that were poorly designed and had the potential to affect the patient flow, access to supplies, and/or patient care [45]. The ability to change a space once bricks, mortar, and drywall are in place is cost prohibitive. Having to implement workarounds in a new environment is frustrating for clinicians expecting things to be done right the first time. Other institutions which plan on using simulation to test the opening of new healthcare facilities can learn from some of these lessons by initiating conversations with senior administration and infrastructure leads early on during the design and construction phase of a new hospital.

Specific metrics, data points, and team evaluations were completed by area, but lacked consistency between areas and ultimately affected the ability to broadly share lessons learned for other projects. Since opening, a formal evaluation and team assessment form has been created and is in use for our proactive approach to move towards a broader and more inclusive integrated system simulation approach of commissioning new spaces and new processes in all provincial hospitals [46]. This new approach includes identifying sponsors and key stakeholders for reporting up and ownership of latent safety threats, identifying metrics and outcome reporting, so that lessons learned do not remain unheard. We have also developed post-session evaluation forms to assist in capturing findings and gaps in the system.

Initial successes for this project started with a shared common goal to deliver the best patient care in a new hospital facility. Administrative leaders’ commitment was essential to ensure a portion of the new practitioners’ orientation time to the site and unit involved simulation exercises. Key stakeholders for each area helped co-design simulations and define what they needed to test and teach. Champions still continue to be drivers of site simulation education today.

## Conclusion

Findings from evaluation and clinical team feedback suggested that simulation exercises were a successful method of testing new processes and systems. Simulations identified latent safety threats which would have otherwise been identified during real patient care after the facility opened. Operational readiness of a newly constructed hospital for emergency, acute, and outpatient care was successfully verified with on-site high realism simulation scenarios. Participant debriefing and survey responses identified several key issues for improvement prior to the opening day. Simulation was also recognized as an optimal training method for staff orientation into a new facility. Newly formed interdisciplinary teams came together for the first time during the simulation, allowing for analysis of team dynamics, role clarity, communication, and leadership.

## Data Availability

Data sharing is not applicable to this article as no datasets were generated or analyzed.

## Abbreviations

AED: Automatic external defibrillator
AHS: Alberta Health Services
CEO: Chief executive officer
CICU: Cardiac intensive care unit
CT: Computerized tomography
ED: Emergency department
EDM: Emergency disaster management
EMS: Emergency medical services
eSIM: Educate Simulate Innovate and Motivate
FM&E: Facilities maintenance and engineering
FMP: Family maternal practice
HCA: Health care aide
HCQA: Health Quality Council of Alberta
ICU: Intensive care unit
LST: Latent safety threats
MRI: Magnetic resonance imaging
NICU: Neonatal intensive care unit
OB: Obstetrics
OR: Operating room
PAC: Pre-admission clinic
PACU: Post-anesthetic care unit
RN: Registered nurse
RRT: Registered respiratory therapist
RT: Respiratory therapy
SHC: South Health Campus
SSH: Society for Simulation in Healthcare

## References

[1] Hoppszallern S, Vesely V, Morgan J. 2016 Hospital Construction Survey [Internet]. 2016 [cited 2018 Sep 19]. Available from: https://www.hfmmagazine.com/articles/1878-2016-hospital-construction-survey

[2] Healthcare GE (2011). Hospital of the future snapshots of success.

[3] Healthcare F. Top healthcare construction projects of 2015 [Internet]. Healthcare Finance News. 2014 [cited 2018 Sep 22]. Available from: https://www.healthcarefinancenews.com/news/top-healthcare-construction-projects-2015-buidling-surges-demand-picks-revista-says

[4] O’Hara S (2014). Planning intensive care unit design using computer simulation modeling: optimizing integration of clinical, operational, and architectural requirements. Crit Care Nurs Q..

[5] Holden RJ, Carayon P, Gurses AP, Hoonakker P, Hundt AS, Ozok AA, Rivera-Rodriguez AJ (2013). SEIPS 2.0: a human factors framework for studying and improving the work of healthcare professionals and patients. Ergonomics..

[6] Carayon P, Schoofs Hundt A, Karsh B-T, Gurses A P, Alvarado C J, Smith M, Flatley Brennan P (2006). Work system design for patient safety: the SEIPS model. Quality and Safety in Health Care.

[7] Harder N (2010). Use of simulation in teaching and learning in health sciences: a systemic review. J Nurs Educ.

[8] Lateef F (2010). Simulation-based learning: just like the real thing. J Emerg Trauma Shock..

[9] Salas E, DiazGranados D, Weaveaver S, King H (2008). Does team training work? Principles for Health Care. Acad Emerg Med.

[10] Posner GD, Clark ML, Grant VJ. Simulation in the clinical setting: towards a standard lexicon. Adv Simul [Internet]. 2017 Sep 20 [cited 2018 Sep 22];2. Available from: https://www.ncbi.nlm.nih.gov/pmc/articles/PMC5806315/10.1186/s41077-017-0050-5 PMC5806315 29450016

[11] Reason J (2000). Human error: models and management. BMJ..

[12] SSH.The Society for Simulation in Healthcare>SSH resources>Dictionary {Internet}2016[cited 2018Sep 19]. Available from : https://www.ssih.org/Dictionary.

[13] Geis GL, Pio B, Pendergrass TL, Moyer MR, Patterson MD (2011). Simulation to assess the safety of new healthcare teams and new facilities. Simul Healthc..

[14] HQCA. Simulation-based mock-up evaluation framework [Internet]. HQCA. 2016 [cited 2018 Sep 19]. Available from: http://hqca.ca/health-care-provider-resources/frameworks/simulation-based-mock-up-evaluation-framework/

[15] Patterson MD, Geis GL, Falcone RA, LeMaster T, Wears RL (2013). In-situ simulation: detection of safety threats and teamwork training in a high risk emergency department. BMJ Qual Saf..

[16] Patterson, MD Blike GT, Nadkarni, VM. In-situ simulation: challenges and results. Advances in Patient Safety: New Directions and Alternative Approaches. 2008: Aug(3) Available from: https://www.ncbi.nlm.nih.gov/books/NBK43682/

[17] Harper MG, Gilbert GE, Gilbert M, Markey L, Anderson K (2018). Simulation use in acute care hospitals in the United States. J Nurses Prof Dev.

[18] Katznelson JH, Wang J, Stevens MW, Mills WA (2018). Improving pediatric preparedness in critical access hospital emergency departments: Impact of a longitudinal in situ simulation program. Pediatr Emergency Care..

[19] Knight P, MacGloin H, Lane M, Lofton L, Desai A, Haxby E, Burmester M. Mitigating latent threats identified through an embedded in situ simulation program and their comparison to patient safety incidents: a retrospective review. Front Pediatr. 2018;5. 10.3389/fped.2017.00281. 10.3389/fped.2017.00281 PMC5810281 29473026

[20] Kurup V, Matei V, Ray J (2017). Role of in-situ simulation for training in healthcare: opportunities and challenges. Curr Opin Anaesthesiol.

[21] Reed DJW, Hermelin RL, Kennedy CS, Sharma J (2017). Interdisciplinary onsite team-based simulation training in the neonatal intensive care unit: a pilot report. J Perinatol.

[22] Couto TB, Kerrey BT, Taylor RG, FitzGerald M, Geis GL (2015). Teamwork skills in actual, in situ, and in-center pediatric emergencies: performance levels across settings and perceptions of comparative educational impact. Simul Healthc.

[23] Ventre KM, Barry JS, Davis D, Baiamonte AA, Wentworth AC, Pietras M (2014). Using in-situ simulation to evaluate operational readiness of a children’s hospital-based obstetrics unit. Simul Healthc..

[24] Adler MD, Mobley BL, Eppich WJ, Lappe M, Green M, Mangold K. Use of simulation to test systems and prepare staff for a new hospital transition. J Patient Saf. 2018. 10.1097/PTS.0000000000000184 26076076

[25] Medwid Kelly, Smith Silas, Gang Maureen (2015). Use of in-situ simulation to investigate latent safety threats prior to opening a new emergency department. Safety Science.

[26] Bender GJ (2011). In-situ simulation for systems testing in newly constructed perinatal facilities. Semin Perinatol.

[27] Hamman WR, Beaubien JM, Beaudin-Seiler BM (2009). Simulation for the training of human performance and technical skills: the intersection of how we will train health care professionals in the future. J Grad Med Educ..

[28] Chan APC (2000). Evaluation of enhanced design and build system a case study of a hospital project. Constr Manag Econ..

[29] Reno K, Grazman D. Operations commissioning: putting new healthcare buildings to the test [Internet]. HCD Magazine. 2014 [cited 2018 Sep 19]. Available from: https://www.healthcaredesignmagazine.com/architecture/operations-commissioning-putting-new-healthcare-buildings-test/

[30] Gardner AK, Ahmed RA, George RL, Frey JA (2013). In situ simulation to assess workplace attitudes and effectiveness in a new facility. Simul Healthc.

[31] Winch P, Khan S, Naguib A, Yates AR, Rice J, Barry N, Tobias JD (2014). Transportation of patients following surgery for congenital heart disease: a process review prompted by the opening of a new hospital. Int J Clin Exp Med.

[32] Santiago C, Zinagano L, Wannamaker K, Bell K, Savedra P, Diston MT, Smith O (2014). Nursing perspectives on designing a space to deliver quality care...dynamics of critical care. Dynamics.

[33] Evans J, Reyers E (2014). Patient room considerations in the intensive care unit. Crit Care Nurs Q.

[34] Ross, R., & Seckman, C. (2016). The challenges of moving from construction to operations: overcoming performance issues with staff orientation and training. Health Facil Manag. 2016: 29(8), 37-40. 30035496

[35] Kerner J, Robert L, Gallo K, Cassara M, DʼAngelo J, Egan A, Simmons JG (2016). Simulation for operational readiness in a new freestanding emergency department: strategy and tactics. Simul Healthc.

[36] Gignon M, Amsallem C, Ammirati C (2017). Moving a hospital: simulation–a way to co-produce safety healthcare facilities. Int J Occup Saf Ergon.

[37] Comeau OY, Armendariz-Batiste J, Baer JG (2017). Preparing critical care and medical-surgical nurses to open a new hospital. Crit Care Nurs Q.

[38] Hauk L (2018). Mock ORs help engage perioperative personnel in design decisions. AORN J.

[39] Eppich W, Cheng A (2015). Promoting Excellence and Reflective Learning in Simulation (PEARLS): development and rationale for a blended approach to health care simulation debriefing. Simul Healthc.

[40] Bajaj K, Meguerdichian M, Thoma B, Huang S, Eppich W, Cheng A (2018). The PEARLS Healthcare Debriefing Tool. Acad Med.

[41] Sawyer T, Eppich W, Brett-Fleegler M, Grant V, Cheng A (2016). More than one way to debrief: a critical review of healthcare simulation debriefing methods. Simul Healthc.

[42] Cheng A, Eppich W, Grant V, Sherbino J, Zendejas B, Cook DA (2014). Debriefing for technology-enhanced simulation: a systematic review and meta-analysis. Med Educ.

[43] Brett-Fleegler M, Rudolph J, Eppich W, Monuteaux M, Fleegler E, Cheng A, Simon R (2012). Debriefing assessment for simulation in healthcare: development and psychometric properties. Simul Healthc.

[44] Halm MA (2005). Family presence during resuscitation: a critical review of the literature. Am J Crit Care.

[45] Eichhorn DJ, Meyers TA, Mitchell TG, Guzzetta CE (1996). Opening the doors: family presence during resuscitation. J Cardiovasc Nurs.

[46] Dubé M, Shultz J, Barnes S, Pascal B, Kaba A. Goals, recommendations, and the how-to strategies for developing and facilitating patient safety and system integration simulations. HERD. 2019. 10.1177/1937586719846586 31060393

